# Trends in nitrate levels in Iowa's community water systems (2000–2022): Characteristics of systems vulnerable to maximum contaminant level exceedances and future regulatory scenarios

**DOI:** 10.1002/jeq2.70189

**Published:** 2026-05-01

**Authors:** S M Samiul Islam, David M. Cwiertny, Ibrahim Demir

**Affiliations:** ^1^ IIHR Hydroscience and Engineering University of Iowa Iowa City Iowa USA; ^2^ Civil and Environmental Engineering University of Iowa Iowa City Iowa USA; ^3^ Department of Chemistry University of Iowa Iowa City Iowa USA; ^4^ River‐Coastal Science and Engineering Tulane University New Orleans Louisiana USA; ^5^ Bywater Institute Tulane University New Orleans Louisiana USA

## Abstract

This study examines trends in nitrate contamination in Iowa's community water systems (CWS) from 2000 to 2022, focusing on the characteristics of CWS that are most vulnerable to elevated nitrate levels and those likely to be impacted by a lower maximum contaminant level (MCL). Using Safe Drinking Water Act (SDWA) compliance data for CWS currently without nitrate removal, we analyzed nitrate levels across CWS types, source water type, well characteristics, and geography. Results show that large CWS serving >100,000 people frequently exceed 5 mg‐N/L due to their reliance on surface water that is vulnerable to non‐point source pollution. Small systems (<10,000 consumers) often exhibit episodic spikes in nitrate, often during spring and early summer, coinciding with fertilizer use and rainfall‐driven leaching. Shallow and pre‐1990 wells were disproportionately affected. Geospatial mapping analysis identified nitrate hotspots in agriculturally intensive regions. A future MCL based on an annual average of 5 mg/L‐N would only affect ∼25 CWS annually, far fewer than those impacted under a scenario where any instance above 5 mg/L‐N would be a violation. These data‐driven findings support future policy for nitrate regulation and drinking water protection.

## Plain Language Summary

This study examined nitrate levels in the drinking water from 2000 to 2022 in Iowa. Nitrate is a chemical commonly used in farm fertilizer and can be harmful to human health at high levels. We studied community water systems, which supply drinking water to towns and cities across Iowa, to understand which systems are most at risk. We found that larger water systems serving >10,000 people often rely on rivers or lakes for drinking water. These surface water sources are more exposed to pollution from nearby agricultural land and show greater changes in nitrate levels over time. Smaller water systems serving fewer than 10,000 people usually depend on groundwater wells. These systems generally had lower average nitrate levels but sometimes experienced short‐term spikes, especially in spring and early summer, after fertilizer is applied to fields and rainfall moves nitrate into the ground. Older and shallower wells found higher nitrate levels than newer or deep wells, likely because older well construction standards provided less protection from contamination. We also examined how different safety rules could affect water systems. If the current legal limit of 10 mg/L were lowered to 5 mg/L and evaluated using yearly averages, only about 25 systems per year would exceed the limit. However, if a single test above 5 mg/L were counted as a violation, many more systems would be affected. Overall, this study shows that nitrate risk in drinking water depends on water source, system size, well construction, and farming activity, and that monitoring and protection strategies should reflect these differences.

**Abbreviations**
CWS: community water systems
EPA: Environmental Protection Agency
GW: ground water
IDNR: Iowa Department of Natural Resources
MCL: maximum contaminant level
PWS: public water systems
SDWA: Safe Drinking Water Act
SW: surface water

## 1 INTRODUCTION

Human activities, such as the use of commercial fertilizers, the application of manure, and sewage treatment, can pollute drinking water sources with nitrate (Craswell, 2021; Ward et al., 2018). Nitrate contamination in drinking water is increasingly recognized as a significant environmental and public health issue globally (Abascal et al., 2022; Shrestha et al., 2025; Ward et al., 2018). This pollutant readily infiltrates soils, contaminating both ground water (GW) and surface water (SW) sources (Khan et al., 2018). The primary anthropogenic sources of nitrate include nitrogen‐based fertilizers, animal manure, wastewater treatment plant discharges, septic systems, and industrial emissions (Moloantoa et al., 2022; Yu et al., 2019). Once introduced, nitrate can enter GW through leaching or reach SWs via runoff, ultimately affecting drinking water supplies and posing health risks to humans and animals (Chen et al., 2016; WHO, 2016). Multiple studies have reported widespread nitrate pollution in the United States, especially in shallow or unconfined aquifers beneath agricultural regions with intensive fertilizer use and well‐drained soils (Burkart & Stoner, 2002; Burow et al., 2010; Hubbard & Sheridan, 2020).

One of the most severe health risks is methemoglobinemia (commonly referred to as blue baby syndrome), a condition that reduces the blood's ability to carry oxygen and can lead to serious illness or fatal outcomes in infants (Johnson, 2019; Knobeloch et al., 2000; Ward et al., 2005). Exposure to elevated nitrate levels during pregnancy has also been associated with adverse fetal outcomes, as nitrate can interfere with maternal oxygen transport and thereby impact fetal development (Manassaram et al., 2010). Moreover, there is growing evidence that chronic exposure to high nitrate levels may be linked to various cancers, including stomach, esophageal, colorectal, and bladder cancers, as well as thyroid dysfunction and other metabolic disorders (Garcia Torres et al., 2022; Ward et al., 2010). The toxicological mechanisms underlying these health effects are still being studied, but they are believed to involve the conversion of nitrate to nitrite, which is subsequently converted to N‐nitroso compounds, potent carcinogens (Seyyedsalehi et al., 2023; Swann, 1975; van Breda et al., 2019).

Iowa, one of the leading agricultural states in the United States, faces a particularly high risk of nitrate contamination in its public water systems (PWS) due to its extensive agricultural activities (Cikmaz et al., 2025; Pollans, 2016; Weigel, 2024). This risk is heightened because the state's economy is heavily reliant on agriculture, with vast areas of land dedicated to crops such as corn and soybeans that require substantial nitrogen fertilization to achieve high yields (ITS, 2020; Jarchow et al., 2012; Tanir et al., 2024). Additionally, the use of synthetic nitrogen fertilizers, while boosting crop productivity, also increases the potential for nitrate runoff (Craswell, 2021; Liu et al., 2021). During precipitation events, nitrate can leach from the soil into GW or be carried by surface runoff into rivers, lakes, and reservoirs, which are often sources of drinking water. Compounding these pathways, the hydrological characteristics of Iowa, including its permeable soils and widespread drainage systems (Craswell, 2021; Keeney & Olson, 1986; Weber et al., 2018), further facilitate the movement of nitrate into water bodies during rainfall and flood events, exacerbating the problem (Roth, 2010). Consequently, improving public awareness and promptly disseminating clear, accessible information on nitrate levels and associated health risks, especially to vulnerable populations, can reduce exposure and strengthen community support for mitigation measures (Demir et al., 2009; Islam et al., 2025; Vald et al., 2026). Examples of mitigation measures include source water protection, agricultural nutrient management practices, blending or alternative water supplies, and treatment technologies such as ion exchange or reverse osmosis.

Although the Safe Drinking Water Act (SDWA) has implemented regulatory mechanisms to monitor and manage nitrate levels in public water sources, nitrate pollution remains an ongoing problem in Iowa (Jones et al., 2018; Wheeler et al., 2015). The US Environmental Protection Agency (EPA) has established a maximum contamination limit (MCL) for nitrate in drinking water at 10 milligrams per liter (mg/L) as nitrogen (Dieter et al., 2018; Dubrovsky et al., 2010). This threshold has been specifically developed to safeguard human health against acute health effects, including methemoglobinemia. Nevertheless, a significant number of PWS in Iowa have reported nitrate levels that are nearing or over this threshold (Levin et al., 2002; Wheeler, 2015). This has raised concerns among public health experts, lawmakers, and the general population, primarily because the US EPA has acknowledged the need for, but has yet to complete, a revised health assessment for nitrate that considers new data on carcinogenicity and developmental effects in their second 6‐year review of drinking water standards in 2010 (Office of the Federal Register, 2010).

This current research provides a comprehensive examination of nitrate levels in the finished water provided by community water systems (CWS) in Iowa from 2000 to 2022. The study aims to identify the types of CWS in Iowa that are most often susceptible to elevated nitrate concentrations. We also conducted an extensive temporal analysis to identify periods characterized by elevated nitrate levels, which may align with distinct agricultural cycles, such as planting and fertilizing seasons, or climatic occurrences, such as intense rainfall and flooding. Moreover, we investigated the spatial distribution of nitrate contamination throughout Iowa's CWS, identifying areas with consistently high concentrations, often referred to as “hot spots,” by mapping nitrate levels geographically. The primary goal of this work was to use this spatial and temporal analysis to assess the vulnerabilities of different CWS based on their size and water source, while also considering those CWS most likely to be impacted by future regulatory scenarios that may result in a stricter MCL for nitrate (e.g., 5 mg/L as N). These results could support the development of information and data visualization systems for enhancing public awareness and decision‐making (Demir & Beck, 2009; Mount et al., 2024).

## 2 METHODOLOGY

### 2.1 Data acquisition

Nitrate concentration data in finished drinking water were obtained from the Iowa Department of Natural Resources (IDNR) for PWS between 2000 and 2022 under SDWA reporting. The dataset included 1838 PWSs, of which 1034 were active CWS. System locations were georeferenced using the reported locations of treatment or distribution facilities provided by IDNR. Only active CWSs were included in the analysis. Sixty‐four systems, including the Des Moines Water Works, were excluded because they employ nitrate removal technologies (e.g., ion exchange and reverse osmosis), as the analysis focused on untreated finished water as a proxy for source water contamination. The dataset was filtered to focus on CWS, excluding non‐transient non‐CWS. Systems were classified by population served as small (≤10,000 residents), medium (10,000–100,000), or large (>100,000). Source water types were classified as GW or SW, and GW systems under the direct influence of SW (n = 5) were excluded. For GW systems, well depth and construction year were obtained from the Well Forecasting System (Ravindran et al., 2025; Sit et al., 2021) to evaluate potential associations between aquifer characteristics and nitrate vulnerability. Nitrate monitoring frequency varied across CWS, ranging from multiple samples per month to less frequent sampling depending on system characteristics and regulatory requirements. All measurements were analyzed by state‐certified or EPA‐approved laboratories using standard methods for nitrate analysis. To address uneven temporal sampling, frequency‐based analytical approaches were applied. Reported nitrate concentrations below analytical detection limits were retained as provided by the IDNR. Summary statistics emphasized medians in addition to means to account for skewed concentration distributions.

### 2.2 Statistical analysis

A multi‐faceted analytical framework was applied to assess nitrate contamination trends, combining descriptive statistics, correlation analysis, temporal decomposition, and comparative statistical testing. Descriptive statistics (mean, median, standard deviation, and range) were calculated separately for GW and SW systems to characterize variability. Associations between nitrate concentrations and system characteristics, including well depth, population served, and source water type, were evaluated using Spearman's rank correlation method. Temporal patterns were examined using moving averages and seasonal‐trend decomposition using LOESS (STL), which separates time series into trend, seasonal, and residual components (Cleveland et al., 1990). Seasonality was defined monthly using calendar months to capture agriculturally relevant periods rather than assuming standard meteorological seasons. Comparisons of nitrate concentrations across system size categories and between GW and SW systems were evaluated using nonparametric statistical tests (e.g., Mann–Whitney U and Kruskal–Wallis tests), which are appropriate for skewed environmental concentration data.

### 2.3 Spatio‐temporal analysis

A GIS‐based spatio‐temporal analysis was conducted to characterize spatial and temporal patterns in nitrate concentrations across Iowa CWS. CWS locations were represented as point features corresponding to reported treatment or distribution facility locations provided by the IDNR. Nitrate concentrations were classified into four categories using a natural breaks (Jenks) approach: low (<1.58 mg/L), moderate (1.58–5 mg/L), high (5–10 mg/L), and extreme (>10 mg/L). The upper‐class threshold was defined to explicitly represent concentrations exceeding the EPA maximum contaminant level (MCL). Annual mean nitrate concentrations for GW and SW systems were mapped separately for each year from 2000 to 2022 to evaluate long‐term spatial patterns. Temporal variability was incorporated by aggregating monthly nitrate observations within each year, allowing seasonal patterns to be visualized consistently across systems with uneven sampling frequency. Spatial analyses were descriptive and exploratory in nature, and no formal spatial statistical trend tests were applied.

### 2.4 Geographic susceptibility and hotspot identification

Geographic susceptibility was evaluated using a kernel density‐based heat map generated in ArcGIS Pro (v3.3.0; Esri) to visualize the spatial concentration of nitrate observations across active CWS. CWS locations were represented as point features, and nitrate concentrations were weighted by magnitude to produce a continuous density surface. In this study, hotspots were defined descriptively as areas where clusters of CWS repeatedly exhibited elevated nitrate concentrations relative to surrounding regions. High nitrate concentrations were defined as values exceeding 5 mg/L as N, a threshold commonly used to indicate increased risk of chronic health effects, while concentrations exceeding 10 mg/L as N were considered extreme. Persistent nitrate contamination refers to CWS locations or clusters where elevated concentrations (≥5 mg/L as N) were observed across multiple years within the study period (2000–2022), rather than as isolated exceedances. The heat map analysis was intended as an exploratory visualization tool to identify spatial patterns of vulnerability rather than as a formal statistical hotspot test.

### 2.5 Data processing and visualization

Prior to analysis, nitrate concentration data were screened for completeness and consistency. Nitrate in finished drinking water is routinely monitored by CWS under the SDWA, with sampling frequency determined by system size, source water type, and historical compliance status. Samples are analyzed by state‐certified or EPA‐approved laboratories using standardized analytical methods and reported to the IDNR.

Records with missing (approximately 3% of observations) nitrate concentration values were excluded from analyses requiring quantitative summaries, while all reported values were retained as provided by the IDNR. No additional statistical outlier removal or value substitution was performed beyond standard regulatory data screening. No statistical imputation of missing values and no post hoc identification or correction of “abnormal” nitrate concentrations were performed beyond standard regulatory quality assurance procedures applied by the reporting agencies. Data visualization techniques were used to summarize and communicate patterns in nitrate concentrations. Boxplots were used to compare distributions across system size categories and source water types, time‐series plots were used to illustrate seasonal and interannual variability, and GIS‐based maps were used to visualize spatial patterns in nitrate concentrations and areas of potential vulnerability.

### 2.6 Case study area

This study focuses on Iowa, a Midwestern state where intensive agricultural land use has significant implications for drinking water quality. Iowa covers approximately 56,272 square miles (145,747 sq. km) and is dominated by row‐crop agriculture, with over 90% of the land area devoted to agricultural production (USDA, 2018). Corn and soybean cultivation account for the majority of this acreage, with corn alone occupying more than half of cultivated cropland and requiring substantial nitrogen fertilizer inputs. These practices promote nitrate leaching to GW and runoff to SW, particularly during spring and early summer precipitation events, contributing to elevated nitrate levels in drinking water sources.

Iowa has approximately 1800 PWS serving a population of about 3.2 million, with nearly 80% of systems relying on GW for drinking water (IDNR, 2022). These systems range from large, centralized utilities serving urban centers such as Des Moines and Cedar Rapids to small, decentralized systems in rural areas. This combination of intensive agriculture and diverse water system characteristics makes Iowa a critical case study for assessing nitrate vulnerability in CWS.

## 3 RESULTS AND DISCUSSION

### 3.1 Community water system overview

Table 1 presents summary statistics for the analysis of nitrate concentrations in finished drinking water for CWS in Iowa that do not use any nitrate removal strategies, and Table 2 shows the CWS classified by population size and well‐depth category. Nitrate concentrations are generally higher in finished drinking water supplied by GW‐dependent CWS, ranging from 0 to 24 mg/L with a mean of 3.13 mg/L, when compared to SW, which ranges from 0 to 10.8 mg/L and has a lower mean value of 2.72 mg/L. The substantially higher maximum nitrate concentration observed in GW‐supplied systems (24 mg/L, exceedingly twice the SDWA MCL) compared to SW systems (10.8 mg/L, slightly above the SDWA MCL) indicates a greater potential for extreme nitrate exceedances in GW sources. This contrast highlights differences in exposure risk to high nitrate levels between source types.

**TABLE 1:** Summary statistics for nitrate concentrations in finished drinking water and system characteristics for community water systems (CWS) in Iowa that do not employ nitrate removal, stratified by source water type.

CWS attribute	Min	Max	Mean	Median	SD
**Ground water systems (GW, *n* = 740**)					
Nitrate concentration (mg/L as N)	0	24	3.1	2.5	3.1
Population served (persons)	25	69,193	11,109	2143	19,636
Well depth (m)	0	1018	63.4	21	113
**Surface water systems (SW, *n* = 20)**					
Nitrate concentration (mg/L as N)	0	10.8	2.7	2.6	1.9
Population served (persons)	25	147,720	55,477	68,753	25,361

*Note*: Values represent minimum (Min), maximum (Max), mean, median, and standard deviation (SD). This table excludes information from the 64 CWS in Iowa that employ a nitrate removal strategy and those using ground water under the direct influence of surface water.Well‐depth statistics are reported only for groundwater‐dependent systems. Well depths originally reported in feet were converted to meters (1 ft = 0.3048 m).

**TABLE 2:** Number of community water systems (CWS) in Iowa included in this study, classified by population size and well‐depth category.

Population category	Ground water systems (*n*)	Surface water systems (*n*)
Small (≤10,000 persons)	655	7
Medium (10,000–100,000 persons)	85	12
Large (>100,000 persons)	0	1
Total	740	20

Well depths for GW‐reliant CWS vary widely, ranging from 0 to 3342 feet (∼1018 m), with an average depth of 209 feet (∼64 m). In addition, populations served by GW systems are generally smaller than those served by SW systems, with mean populations of 11,109 and 55,477, respectively. These differences have implications for system‐level capacity, as smaller, GW‐dependent communities may face greater challenges in managing episodic high nitrate concentrations compared to larger SW systems. Among GW‐dependent systems, the majority served small populations and relied on shallow to intermediate‐depth wells, whereas SW systems more frequently served medium to large populations.

**FIGURE 1:**
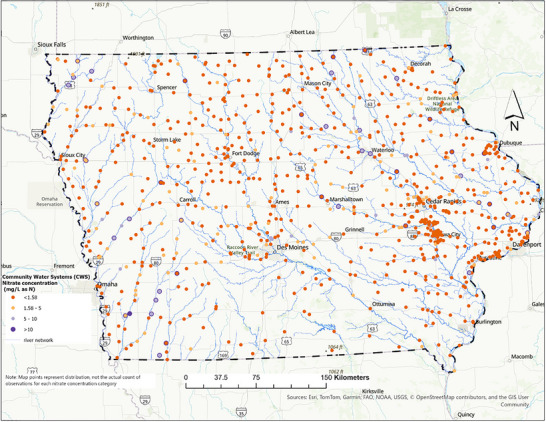
Spatial distribution of nitrate concentrations in finished drinking water across community water systems (CWS) in Iowa. Each point represents a CWS, and point color denotes the nitrate concentration category (mg/L as N). Major river networks are shown for hydrologic context.

Figure 1 illustrates the spatial distribution of CWS across Iowa, categorized by mean nitrate concentrations (mg/L as N) from 2000 to 2022. Each point represents a system's 22‐year average, with color gradations denoting concentration ranges based on a natural breaks classification. Thresholds at 5 and 10 mg/L‐N correspond to concentrations linked to adverse health outcomes, including cancer and birth defects (Ward et al., 2018).

The spatial pattern shows that communities with elevated nitrate levels cluster in northeastern Iowa and along the western border, where moderate to high concentrations are most persistent. These areas represent potential nitrate “hotspots” that require targeted interventions, such as advanced removal technologies. In contrast, central and southeastern Iowa are dominated by blue points, reflecting consistently lower nitrate levels in finished drinking water.

### 3.2 Temporal analysis

#### 3.2.1 Seasonal variability

**FIGURE 2:**
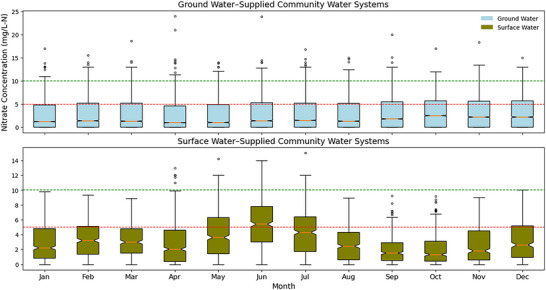
Seasonal variability in monthly nitrate concentrations (mg/L) for community water systems (CWS) not using nitrate removal and relying on ground water (blue) and surface water (green) from 2000 to 2022. The red and green dashed lines represent the 5 and 10 mg/L‐N reference points, respectively, for elevated nitrate levels that may be of concern for chronic health effects.

Monthly nitrate concentration distributions for CWS relying on GW and SW from 2000 to 2022 are shown as box‐and‐whisker plots in Figure 2. A red dashed line indicates the 5 mg/L‐N reference level. This reference level is used because epidemiologic evidence summarized in a comprehensive review by Ward et al. (2018) indicates that associations between drinking water nitrate and adverse chronic health outcomes, including cancer and adverse birth outcomes, are more frequently observed at concentrations above approximately 5 mg/L‐N, even though the current EPA MCL is 10 mg/L‐N.

Consistent with this health‐based benchmark, recent Iowa‐focused drinking water assessments have also adopted the 5 mg/L‐N threshold to identify systems potentially vulnerable to nitrate contamination. For example, Mantey et al. (2025) evaluated 871 Iowa PWS over the 2012–2022 period and classified systems exceeding this level as high‐risk, finding that approximately 2.5% of systems consistently surpassed 5 mg/L‐N and disproportionately affected socioeconomically disadvantaged and racially marginalized communities.

Across the state, SW (green boxes) shows apparent seasonal increases in nitrate concentrations during May, June, and July. Median concentrations rise closer to or above the 5 mg/L‐N threshold during these months, with an overall wider distribution of values, suggesting higher variability. This behavior is consistent with increases in SW nitrate during the planting and growing seasons, presumably due to runoff from land‐applied fertilizers (e.g., commercial anhydrous ammonia and livestock manure), given that 70% of Iowa's land is used for corn‐soybean production (USDA, 2018). For example, prior work has demonstrated that runoff from livestock manure, commonly applied as a supplemental nutrient source for crops, can be a significant contributor to SW nitrate concentrations in watersheds with a large number of animal units (Jones et al., 2019).

In contrast, nitrate levels in GW‐supplied CWS appear more stable throughout the year, with median concentrations remaining below the 5 mg/L‐N reference level in most months. However, occasional outliers indicate sporadic nitrate spikes in April and other months associated with the regional corn (maize) and soybean production cycle. In Iowa, nitrogen fertilizers and manure are commonly applied in early spring (March–April) prior to planting and, in some cases, during the post‐harvest fall period, increasing the potential for nitrate leaching to shallow aquifers. These outliers may therefore reflect highly vulnerable GW sources, such as shallow or older wells influenced by localized recharge following fertilizer application. For example, elevated nitrate concentrations observed in June (Figure 2) may indicate a delayed GW response to spring nutrient applications combined with early‐season precipitation.

**FIGURE 3:**
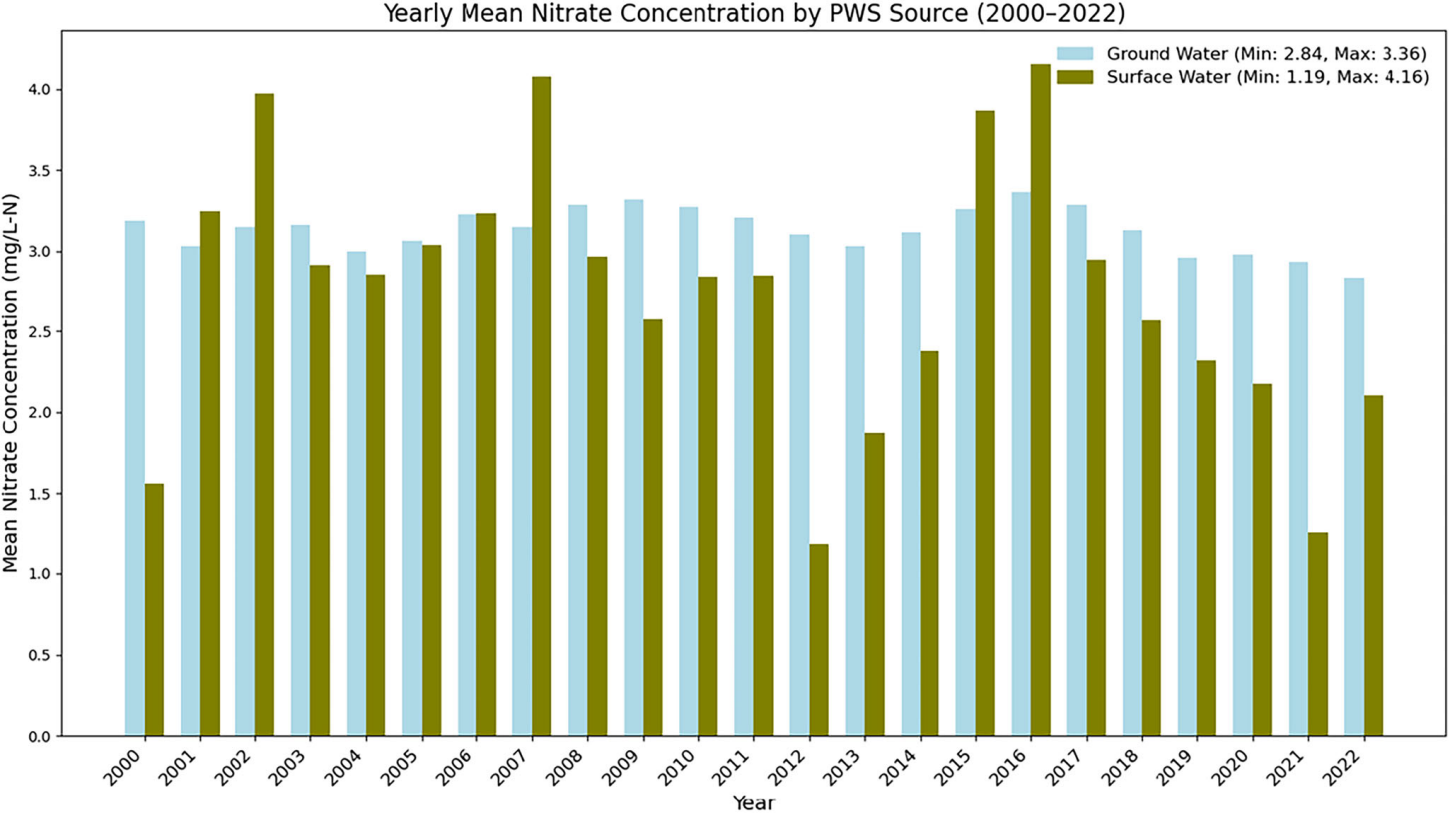
Yearly mean drinking water nitrate concentrations for community water systems (CWS) not using nitrate removal and relying on ground water (blue) and surface water (green) sources during the 2000–2022 period.

Figure 3 shows annual mean values for drinking water nitrate concentrations (mg/L‐N) for CWS using either GW or SW sources from 2000 to 2022. Yearly trends largely align with those from seasonal observations previously discussed in the monthly data (see Figure 2). CWS relying on SW exhibit substantially greater interannual variability in nitrate concentrations than GW‐dependent systems, with annual mean values ranging from approximately 1.17–4.2 mg/L during the study period. In contrast, GW‐reliant systems show comparatively stable annual mean nitrate concentrations, varying within a narrower range of approximately 2.8–3.4 mg/L. Notably, lower SW nitrate concentrations in 2012 may reflect reduced runoff during drought conditions, which limited nitrate transport from agricultural fields into SW supplies.

Years with elevated SW nitrate concentrations often coincide with periods of above‐average precipitation documented in Iowa; however, this relationship is described qualitatively in this study. Notably, significant peaks in drinking water nitrate concentrations for SW systems were observed in 2002, 2003, 2015, and 2016. These years are documented as having higher‐than‐average precipitation rates, aligning with increased runoff events. For example, NOAA's Climate Data Online provides detailed records that confirm these years experienced substantial rainfall, likely enhancing nitrate runoff from agricultural lands (National Oceanic and Atmospheric Administration, 2023a). Additionally, the NOAA Storm Events Database lists specific storm events during these years that could have contributed to elevated nitrate levels by increasing runoff (National Oceanic and Atmospheric Administration, 2023b). Formal statistical correlation analyses between precipitation and nitrate concentrations were not conducted, and not all wet years were associated with increased drinking water nitrate levels. These observations nonetheless highlight the sensitivity of SW systems to hydrologic conditions that influence non‐point source nutrient transport.

### 3.3 Nitrate levels across different CWS sizes

**FIGURE 4:**
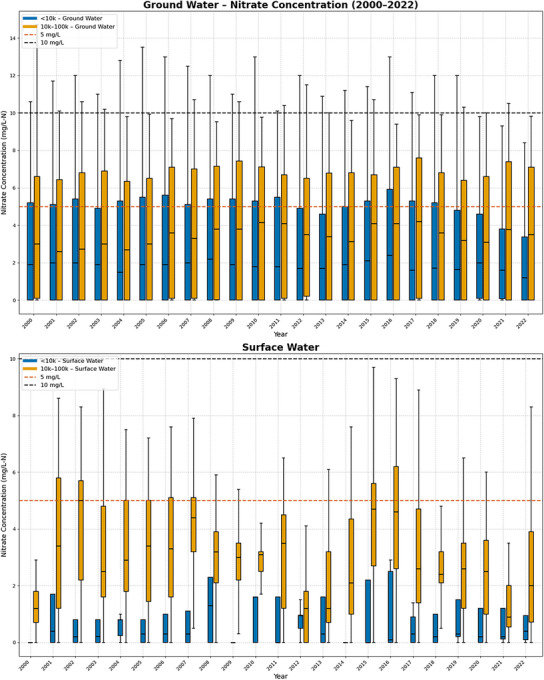
Nitrate concentrations for community water systems (CWS) in Iowa not using nitrate removal and relying on ground water (top) and surface water (bottom) as a function of system size: small (<10,000) and mid‐sized (10,000–100,000). Box plots show the median, interquartile range, and variability from 2000 to 2022. Dashed lines mark the 5 mg/L‐N reference level and the 10 mg/L‐N US Environmental Protection Agency (EPA) maximum contaminant level.

Figure 4 shows nitrate concentrations in GW and SW grouped by population size as small (< 10,000; *n* = 662) and mid‐sized (10,000–100,000; *n* = 97). Iowa has no GW‐dependent CWS serving >100,000 people and only one SW‐supplied system in this category that does not employ nitrate removal (Cedar Rapids). Consequently, large systems (>100,000) were not included in the statistical comparisons, and results for Cedar Rapids are described qualitatively. The Cedar Rapids SW system exhibits greater interannual variability in nitrate concentrations than smaller systems, consistent with patterns observed for SW systems overall.

Across small and mid‐sized CWS, GW nitrate concentrations remain relatively consistent from year to year, with limited fluctuations in median values, consistent with the results in Figure 3. Mid‐sized CWS generally exhibit slightly higher nitrate concentrations than smaller systems, including several years with significant fractions of tests above the 5 mg/L‐N reference level and several over the US EPA MCL of 10 mg/L‐N. Analysis of mean concentrations suggests this pattern, with mid‐sized systems showing a range of mean nitrate concentrations from 3.2 mg/L to 4.2 mg/L‐N, compared to smaller systems, which range from 2.1 mg/L to 3.1 mg/L‐N. These differences are reflected in the interquartile ranges and upper whiskers shown in the boxplots, which illustrate greater variability in nitrate concentrations among mid‐sized GW systems relative to small systems.

In contrast, there are apparent differences in the year‐to‐year variability of drinking water nitrate as a function of system size for CWS relying on SW (Figure 4, bottom). Mid‐sized (10,000–100,000) and large (>100,000) SW systems tend to exhibit higher median nitrate concentrations and wider interquartile ranges than small SW systems. For example, mid‐sized systems showed mean nitrate concentrations ranging from 0.9 mg/L to 2.7 mg/L‐N, while large systems demonstrated even higher ranges, from 1.6 mg/L to 5.6 mg/L‐N. However, the limited number of SW systems, particularly in the large system category, introduces substantial uncertainty. Accordingly, these differences are presented as descriptive patterns based on observed variability rather than as statistically significant differences.

#### 3.3.1 Susceptibility analysis

**FIGURE 5:**
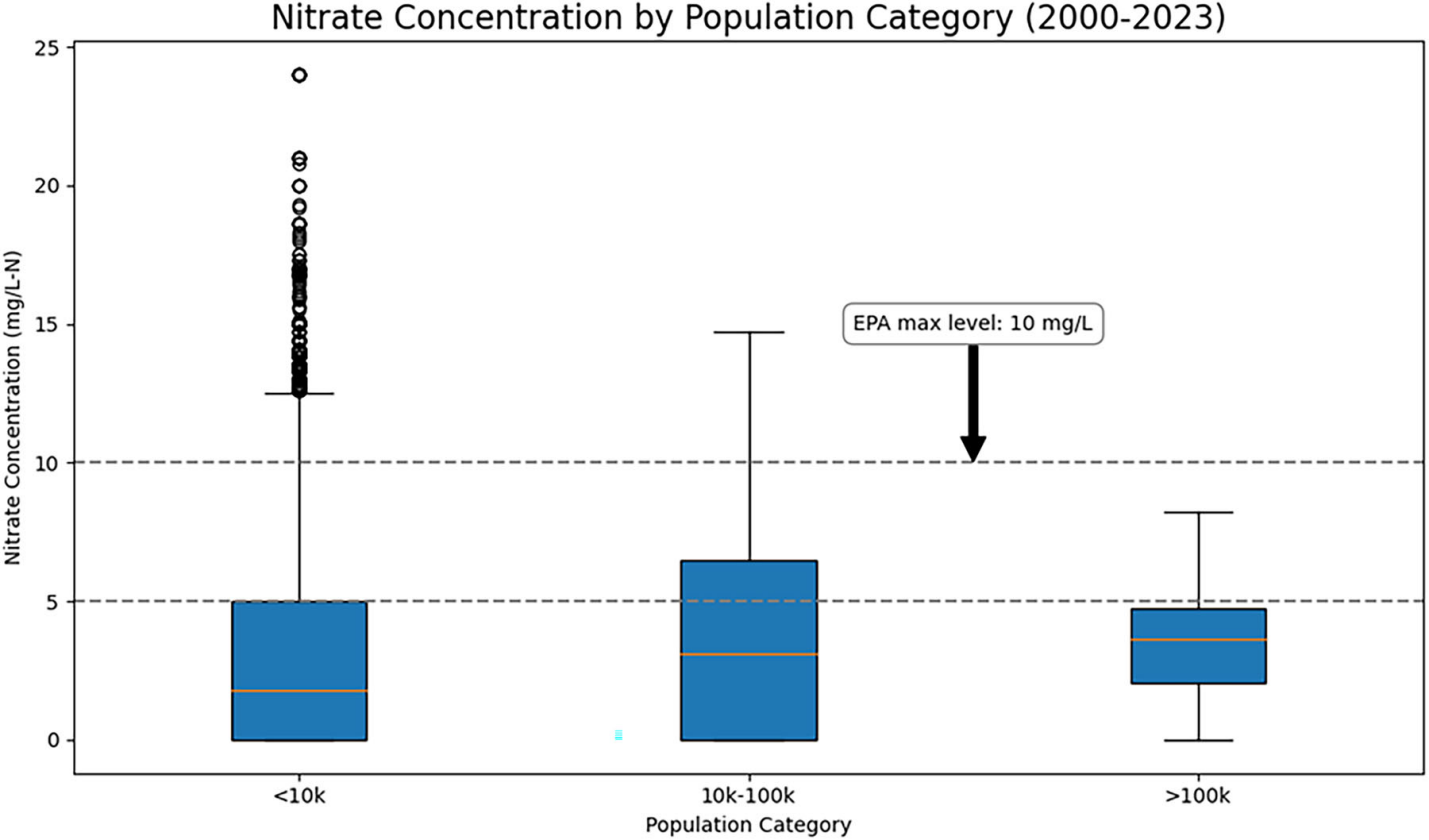
Boxplot of nitrate concentrations (2000–2022) across community water systems categorized by population served (< 10,000; 10,000–100,000; > 100,000). The > 100,000 category represents data from a single large surface water system (Cedar Rapids, Iowa), which does not utilize nitrate removal and is included for contextual comparison only. As this category reflects an *n*‐value of one, no statistical inference should be drawn.

Susceptibility to nitrate contamination was assessed based on the proportion of concentrations exceeding the US EPA's MCL of 10 mg/L and a secondary threshold of 5 mg/L that would be more protective of public health. Figure 5 illustrates the distribution of nitrate concentrations across three population categories: small systems (<10,000), mid‐sized systems (10,000–100,000), and large systems (>100,000), regardless of the source water type. The boxplot highlights median values, variability, and outliers for each category, regardless of source water type.

Small systems (<10,000) show the widest variability, with numerous outliers exceeding 5 and 10 mg/L thresholds. These systems often serve rural communities with limited resources for acquiring advanced nitrate treatment, leaving them vulnerable to contamination. In contrast, mid‐sized systems (10,000–100,000) have higher median nitrate levels, with several results exceeding the 5 and 10 mg/L‐N thresholds. However, mid‐sized CWS have far fewer extreme outliers, perhaps reflecting less vulnerability in response to stressors that tend to exacerbate nitrate pollution. The third category (>100,000), shown in Figure 5, reflects data from the only large community water system included in this analysis (Cedar Rapids), which does not utilize nitrate removal and is shown for contextual comparison only. The large system generally shows lower nitrate levels than the small and mid‐sized systems, and there are no MCL exceedances.

Disparities in nitrate contamination susceptibility pose ongoing and future challenges for Iowa's small systems. As Rauh and Hughes (2024) note, these systems often lack the financial, technical, and personnel resources to maintain safe water supplies, leaving them especially vulnerable to worsening nitrate contamination. Targeted measures—such as subsidies for treatment technologies and stronger source protection—are essential to safeguard public health in these communities.

**FIGURE 6:**
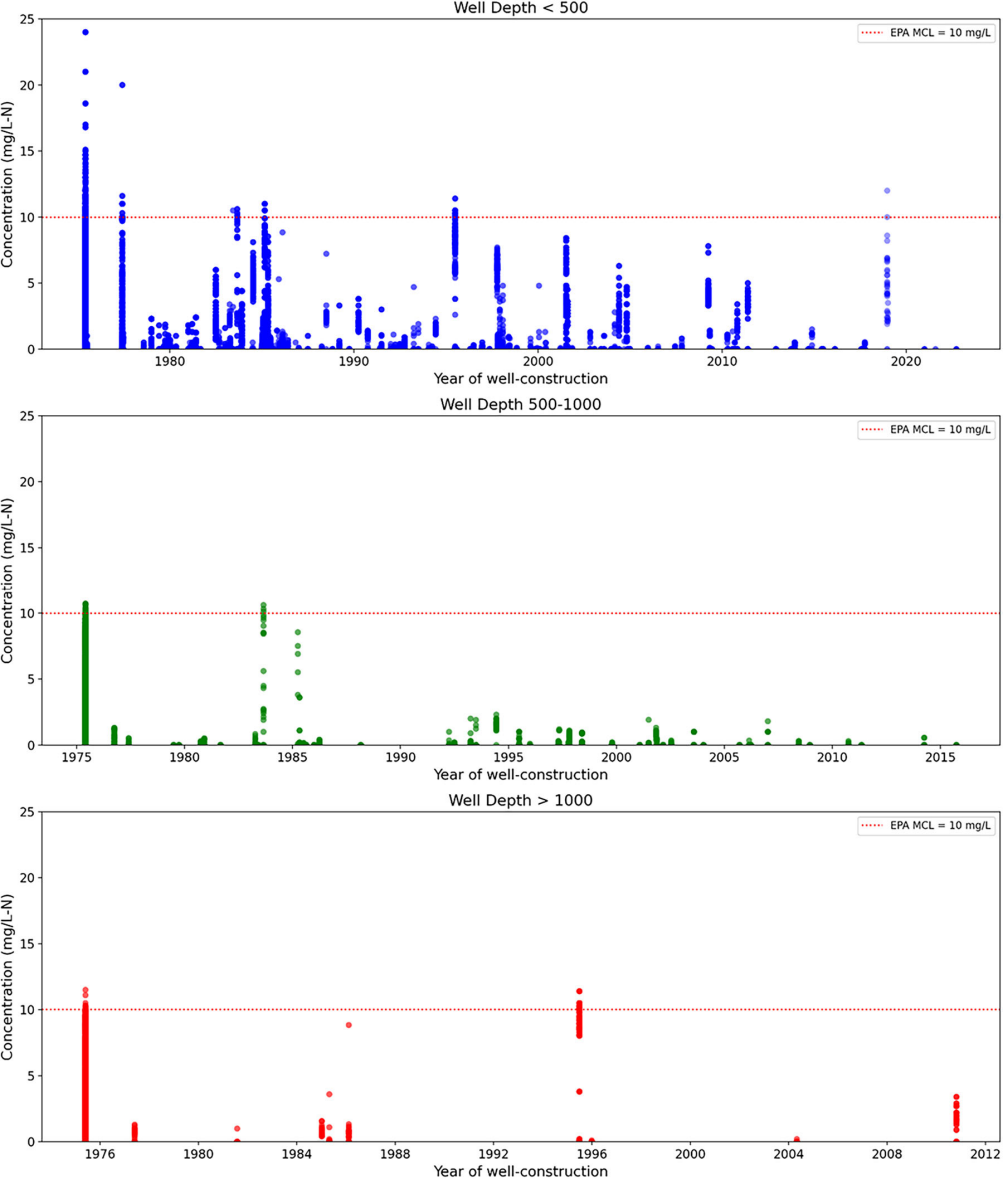
Scatter plots of nitrate concentrations in community water system wells, grouped by depth: shallow (<500 ft), intermediate (500–1000 ft), and deep (>1000 ft). Each point represents the nitrate concentration associated with a well, plotted by the year of well construction. The red dashed line indicates the Environmental Protection Agency (EPA's) maximum contaminant level of 10 mg/L‐N. Note: Concentrations are not averaged; each dot represents an individual observation and x‐axis ranges differ among panels due to differences in construction‐year availability across well‐depth categories. Axes are not aligned to avoid implying data coverage where observations do not exist.

For GW‐sourced CWS, we examined how nitrate concentrations vary with source water infrastructure age and well characteristics. Figure 6 plots nitrate levels against well depth (<500 ft, 500–1000 ft, >1000 ft) by year of construction. Shallow wells (<500 ft) consistently show higher nitrate, often exceeding the 5 mg/L‐N reference and approaching or surpassing the EPA's 10 mg/L‐N MCL. This pattern is most evident in wells built before 1990, suggesting a shift toward deeper wells that are less prone to contamination. Wells constructed after 2010 show lower nitrate levels across depths, reflecting improved standards, enhanced protection, and reliance on less vulnerable sources.

**FIGURE 7:**
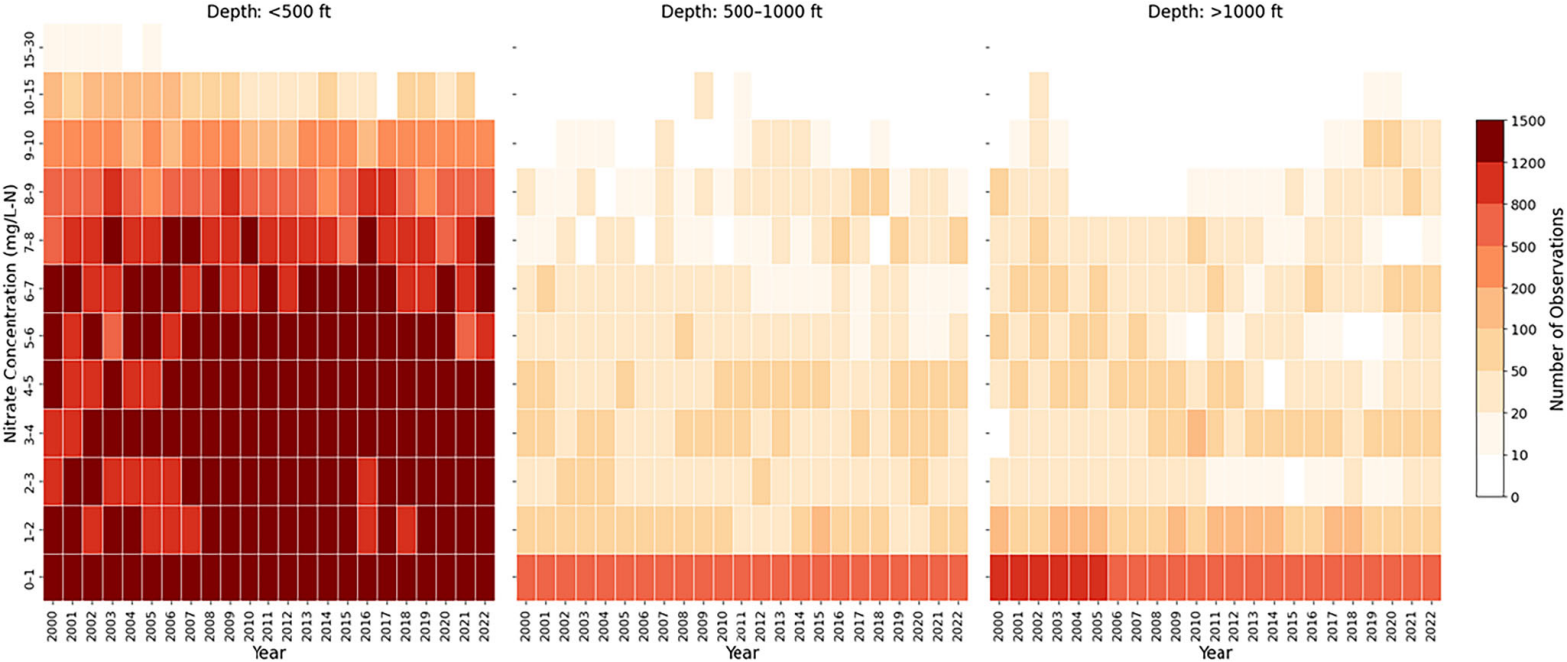
Heatmaps of the frequency distribution of nitrate concentrations for community water systems (CWS) not using nitrate removal and relying on ground water across three well depth categories: shallow (<500 ft), intermediate (500–1000 ft), and deep (>1000 ft) wells. The intensity of color denotes the quantity of observations in each bin annually, with darker red hues indicating a higher frequency of observations.

Figure 7 shows heatmaps of nitrate concentrations in GW across shallow (<500 ft), intermediate (500–1000 ft), and deep (>1000 ft) wells. Color intensity reflects the annual frequency of observations, with darker reds indicating higher counts. Shallow wells consistently exhibit elevated frequencies in the 2–10 mg/L‐N range, often near or above the EPA's 10 mg/L MCL. Intermediate and deep wells record far fewer high‐nitrate events, though this partly reflects lower sampling. Across the 23‐year period, shallow wells show persistent nitrate dominance, underscoring their greater vulnerability.

### 3.4 Geographic analysis

#### 3.4.1 Hot spot identification

**FIGURE 8:**
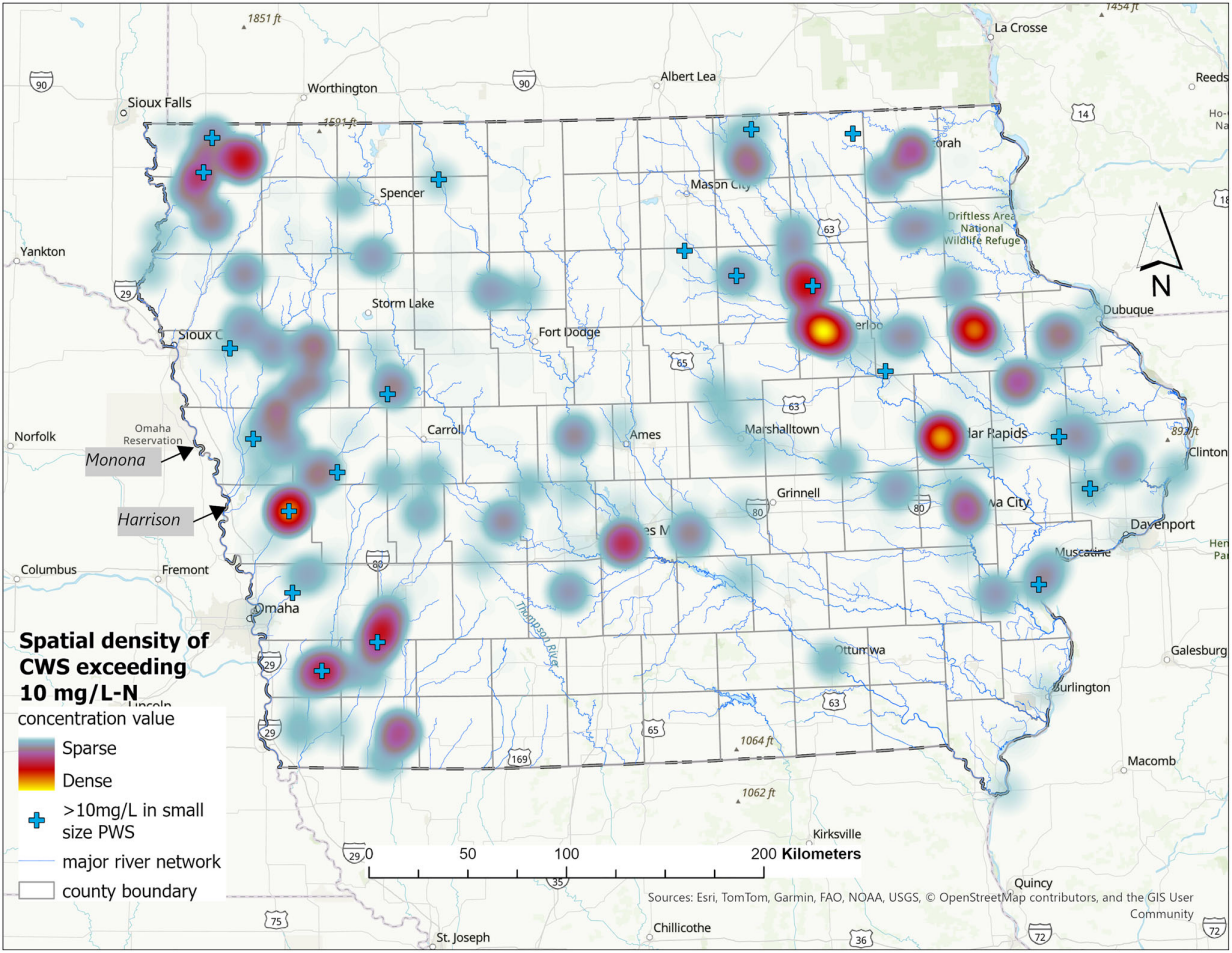
Spatial density of community water systems (CWS) without nitrate removal that recorded nitrate concentrations exceeding 10 mg/L–N in Iowa (2000–2022). The heat map represents spatial concentration of exceedances, with red indicating higher density. Symbols indicate locations of affected CWS of all sizes.

For CWS currently without nitrate removal, GIS‐based spatial analysis revealed recurring areas of elevated nitrate concentrations across Iowa during the 2000–2022 period (Figure 8). Heat maps were generated in ArcGIS Pro (version 3.3.0; Esri) using the Point Density‐based heat map symbology for point layers, which aggregates the spatial distribution of active CWS and applies a continuous color gradient to visualize relative nitrate concentration intensity. Denser areas with higher nitrate concentrations are displayed in red, while lighter colors represent lower densities, effectively highlighting spatial distribution patterns and areas of concern.

The heat map analysis highlights clustering of nitrate exceedances near several major CWS, including Cedar Rapids and Waterloo, as well as broader areas of western Iowa. Cedar Rapids (∼136,000 population, 2023 census) and Waterloo (∼67,000) are located within the agriculturally dominated Cedar River watershed, where extensive row‐crop agriculture (corn and soybeans) and subsurface tile drainage promote nitrate transport to surface and GW following precipitation events (USDA, 2018).

Elevated clustering is also evident in western Iowa within the Missouri River watershed, where intensive row‐crop production and high‐density livestock operations are common. Counties such as Monona and Harrison report some of the highest animal unit densities in the state (Iowa DNR, 2022), and fertilizer application rates in this region are among the highest statewide (USDA, 2018). Rather than representing a uniquely dominant hotspot, western Iowa exhibits nitrate exceedance patterns comparable to those observed near eastern and central Iowa systems, including areas surrounding Waterloo and the Des Moines metropolitan region.

Figure 8 further illustrates that nitrate exceedances are not confined to large systems. Smaller CWS, shown as individual markers, occasionally exceed regulatory thresholds, consistent with patterns observed in Figure 5. Together, these results indicate that nitrate vulnerability is spatially widespread across Iowa, with clustering driven by regional agricultural intensity, hydrogeologic conditions, and water source characteristics.

### 3.5 Potential impact of lowering the nitrate MCL

To consider the number of water systems in Iowa that would be impacted by a lower nitrate MCL of 5 mg/L‐N, we considered two hypothetical regulatory scenarios. The first is a new MCL as a hard cap, such that any instance of monitoring producing a water sample with nitrate above 5 mg/L‐N would produce a health‐based violation. The second considered an annual average, where a health‐based violation would only be triggered if the annual average (considered herein to be a calendar year) for a water system exceeded 5 mg/L‐N. This second scenario is consistent with the current EPA regulatory framework for drinking water contaminants such as total trihalomethanes (TTHMs) and haloacetic acids (HAA5), which are regulated using a locational running annual average that is compared against their respective MCLs under the SDWA (U.S. Environmental Protection Agency, 2006).

In the first scenario, which assumes a hard cap of 5 mg/L‐N, we limited our analysis to the last 10 years (from 2012 to 2022) and examined the percentage of tests for each CWS that exceeded the 5 mg/L‐N limit. Over these 10 years, we found that 100 CWS in Iowa had at least one reported nitrate concentration above 5 mg/L‐N. We identified 16 CWS that had over 90% of all reported nitrate values exceeding 5 mg/L‐N, indicating that these systems would persistently violate a 5 mg/L‐N standard on a yearly basis. Likewise, 43 CWS in Iowa had at least 50% of reported nitrate values above 5 mg/L‐N, and these systems would likely also be in near‐constant violation of the hard‐cap MCL at 5 mg/L‐N. These are generally smaller CWS, with the 100 systems reporting a nitrate level above 5 mg/L‐N within the last decade, serving a total of 735,555 consumers (roughly 23% of Iowa's population). These are systems and consumers that would likely need to seek out advanced nitrate treatment, which would need to operate almost year‐round, or an alternative source water supply in the event of a stricter nitrate MCL of 5 mg/L‐N.

Under the second scenario, which relies on an annual average of nitrate concentrations relative to the 5 mg/L‐N threshold, we find that far fewer CWS would be impacted by new regulations. Examining the annual average in 2022, for instance, we observe that only 23 CWS had averages above 5 mg/L‐N (although three additional systems had averages above 4.8 mg/L‐N). In years with higher SW nitrate levels, typically corresponding to wetter years in Iowa, more systems would be at risk of violation. For example, in 2016, which is regarded as a year with some of the highest SW nitrate levels in recent Iowa history, 53 CWS would have exceeded or met 5 mg/L‐N as their annual average based on compliance testing (with another six CWS at or above 4.8 mg/L as N). Thus, while this scenario results in fewer CWS that would violate a stricter SDWA standard based on a hard cap of 5 mg/L‐N, the number of systems in violation could reasonably be expected to vary considerably from year to year.

Because a new, stricter drinking water standard would likely address chronic exposure risks associated with nitrate in drinking water, using an annual average that better corresponds to sustained, long‐term exposure would likely represent a more reasonable approach. Moreover, it would result in a smaller number of systems being affected by a stricter nitrate MCL and thus requiring the implementation of treatment and/or the acquisition of a new source water supply. This may, in turn, improve the outcomes of economic analyses that would need to be conducted to justify the cost of a new nitrate regulation, thereby increasing the likelihood that a new, lower nitrate MCL could be finalized and implemented.

## 4 CONCLUSION

The present analysis of nitrate concentrations in Iowa's CWS (2000–2022) reveals key spatial, temporal, and infrastructural drivers of contamination. Nitrate levels varied widely (0–24 mg/L; mean 3.17 mg/L) and were unevenly distributed, shaped by population size, water source, well depth, and geography. Geospatial hotspot analysis identified consistently elevated concentrations around Cedar Rapids, Waterloo, and western Iowa, reflecting the influence of land use, source water type, and the need for stronger localized management.

The analysis suggests that GW systems exhibit patterns consistent with infrastructure effects, with older wells (pre‐1990) generally associated with higher nitrate concentrations and newer wells (post‐2010), presumably built with a better understanding of nitrate‐vulnerable aquifers, associated with lower levels. SW systems showed greater variability than GW systems, consistent with increased sensitivity to watershed‐scale processes such as agricultural runoff. Seasonal increases observed in spring and early summer align with known fertilizer application periods and rainfall patterns, indicating that these periods may warrant enhanced monitoring.

Although smaller CWS generally reported lower nitrate, some rural systems exhibited dangerously high levels, posing risks to resource‐limited communities lacking treatment capacity. These findings call for targeted assistance, funding, and advanced treatment to ensure equitable access to safe drinking water.

A recent study by Mantey et al. (2025) examines long‐term nitrate levels in Iowa PWS between 2012 and 2022 with a focus on sociodemographic disparities in potential exposure. While that work provides important insight into exposure inequities over the past decade, the present analysis differs in several keyways. First, we extend the temporal window to 2000–2022, enabling identification of longer‐term trend dynamics not captured in a 10‐year window. Second, we explicitly incorporate system‐level characteristics such as source water type, well depth, and community size, allowing us to assess infrastructure vulnerability in addition to exposure patterns. Third, our study integrates seasonal and physiographic analyses and frames findings in a regulatory context by comparing alternative compliance scenarios, providing a broader perspective on nitrate risk and its implications for water management. Together, these differences demonstrate the unique contributions of the current work to the literature on nitrate contamination in drinking water.

The implications are significant for public health and water management. Elevated nitrate in drinking water is linked to methemoglobinemia and possible cancer risks. Identifying vulnerable populations and high‐risk regions supports interventions such as stricter fertilizer controls, advanced treatment technologies, and expanded surveillance. For larger systems, investment in monitoring and treatment upgrades is essential, while small rural systems require financial and technical assistance to overcome capacity limitations.

Managing nitrate in Iowa's CWS represents a complex challenge at the intersection of environmental conditions, infrastructure, and community capacity. By identifying both high‐risk regions and vulnerable populations, the present analysis offers evidence to guide policymakers, public health officials, and water managers in developing targeted strategies. Strengthening monitoring, investing in treatment technologies, and tailoring assistance for small resource‐limited systems will be essential steps toward ensuring safe and reliable drinking water across urban and rural communities alike.

This analysis is primarily descriptive and pattern‐based, and several limitations should be acknowledged. While population size, water source, well depth, seasonality, and geographic location were each associated with nitrate variability, these factors are not independent and likely interact or confound one another. For example, shallow wells are more common in agriculturally intensive regions, and smaller systems are often located in rural areas with limited treatment capacity. As a result, this study does not estimate the independent magnitude of effect of individual drivers or establish causal relationships. A multivariate statistical framework, such as regression or mixed‐effects modeling, would be required to disentangle correlated influences and quantify relative contributions while controlling confounding factors. Such analyses were beyond the scope of the present study, which aimed to characterize long‐term spatial, seasonal, and infrastructural patterns in nitrate occurrence across Iowa's CWS. Future work integrating formal causal or multivariate approaches would strengthen inference and support prioritization of intervention strategies.

## AUTHOR CONTRIBUTIONS

**S M Samiul Islam**: Conceptualization; data curation; formal analysis; investigation; methodology; visualization; writing—original draft; writing—review and editing. **David M. Cwiertny**: Conceptualization; funding acquisition; project administration; supervision; validation; writing—review and editing. **Ibrahim Demir**: Funding acquisition; project administration; validation; writing—review and editing.

## CONFLICT OF INTEREST STATEMENT

The author declare no conflicts of interest.
